# Successful Therapy for Myocarditis Concomitant With Complete Heart Block After Pembrolizumab Treatment for Head and Neck Squamous Cell Carcinoma: A Case Report With Literature Review

**DOI:** 10.3389/fcvm.2022.898756

**Published:** 2022-05-12

**Authors:** Lina Su, Chuanfen Liu, Wenjie Wu, Yuxia Cui, Manyan Wu, Hong Chen

**Affiliations:** ^1^Department of Cardiology, Peking University People's Hospital, Beijing, China; ^2^Department of Oral and Maxillofacial Surgery, Peking University School and Hospital of Stomatology, Beijing, China

**Keywords:** immune checkpoint inhibitors, myocarditis, complete atrioventricular (AV) block, head and neck squamous cancer cells (HNSCC), global longitudinal peak strain

## Abstract

Immune checkpoint inhibitors (ICIs) have revolutionized cancer therapy over the past decade. Despite their beneficial effects on treating numerous types of tumors, cardiotoxicity resulting from ICIs is a rare side effect but a concerning one due to its high mortality rate. We herein describe a case of an 80-year-old woman with recurrent head and neck squamous cell cancer (HNSCC), who presented with myocarditis complicated by complete atrioventricular block (CAVB) after second infusion of pembrolizumab. After quickly ruling out myocardial infarction and viral myocarditis, the strong relationship between the onset time and pembrolizumab therapy suggested that ICI-induced myocarditis was the most possible diagnosis. Though CAVB frequently presents with fulminant myocarditis in the setting of ICI-related cardiotoxicity, the patients kept a stable hemodynamic status and had normal myocardial function with just a slightly low global longitudinal strain (GLS) at−16.4%, which implied myocardial injury but was highly related to good prognosis based on the existing literature. Besides, elderly patients are vulnerable to adverse outcomes of steroid therapy, notably opportunistic infections. To balance beneficial effects and adverse effects of immune suppression, she accepted high-dose steroids without pulse methylprednisolone. Excitingly, she had a dramatic clinical and laboratory improvement, and heart block quickly returned to normal sinus rhythm. Another interesting finding was that the patient's tumor remained stable during the half-year follow-up from the termination of immunotherapy. Besides, we here firstly review previously reported cases in terms of their clinical characteristics and prognosis of ICI-induced myocarditis with CAVB, in particular the reversibility of heart block. In conclusion, ICI-induced myocarditis can be life-threatening and it therefore warrants efforts to increase awareness, facilitate early detection, and initiate prompt intervention. Importantly, CAVB secondary to ICIs-induced myocarditis may not always present with fulminant myocarditis and more than 50% of these surviving patients might recover to normal sinus rhythm. For patients with ICI-induced myocarditis with contraindication for cardiac magnetic resonance (CMR), speckle-tracking echocardiography is a reliable and sensitive alternative to CMR for detecting myocardial injury, and GLS may be an important prognostic indicator.

## Introduction

Immune checkpoint inhibitors (ICIs) have transformed the treatment landscape of many different types of cancers in recent years (1). Pembrolizumab, a humanized monoclonal IgG4 antibody, binds to programmed death receptor-1 (PD-1) and blocks its interaction with programmed death ligand-1 (PD-L1), thereby triggering the patients' immune system to recognize and combat cancer cells (2). Based on the survival results from the phase III clinical trial Keynote 048, pembrolizumab has been approved as the preferred first-line treatment for patients with recurrent or metastatic head and neck squamous cell carcinoma (HNSCC) who have no surgical or radiotherapeutic option (3). Despite favorable benefits, immune-related adverse events (irAEs) have occured in 70–90% of patients treated with ICIs (2). The most common irAEs have been found in the skin, colon, liver, lungs, pituitary gland, and thyroid (2). Cardiotoxicity resulting from ICIs is uncommon but potentially fatal. The incidence of ICI-induced myocarditis ranges from 0.01 to 1.1%, with a mortality rate of up to 50% (4). However, due to the low incidence of cardiac irAEs, data on presentation, diagnosis, treatment, and outcomes are limited (1). We herein present a case of recurrent HNSCC who presented with myocarditis complicated by complete atrioventricular block (CAVB).

## Case Presentation

An 80-year-old woman was diagnosed with primary intraosseous squamous cell carcinoma of the mandible and underwent radical resection in December 2020. Adjuvant therapy was recommended, while the patient could not tolerate chemotherapy or radiotherapy. Five months after the primary surgery, the right mandibular mass recurred and was confirmed as recurrent HNSCC. Immunohistochemistry showed a PD-L1 expression level with a tumor proportion score of 15% and a combined positive score of 15. Given the patient's advanced age and vulnerability, radical resection and adjuvant chemoradiotherapy were not advantageous. Scholars have recommended PD-1 inhibitor monotherapy or PD-1 inhibitors combined with epidermal growth factor receptor (EGFR) inhibitors for the treatment of recurrent HNSCC with no surgical or chemoradiotherapeutic option (5, 6). The patient received pembrolizumab (200 mg) and nimotuzumab (200 mg), with an intravenous delivery every 3 weeks. She had no previous history of cardiovascular diseases.

**Figure 1:**
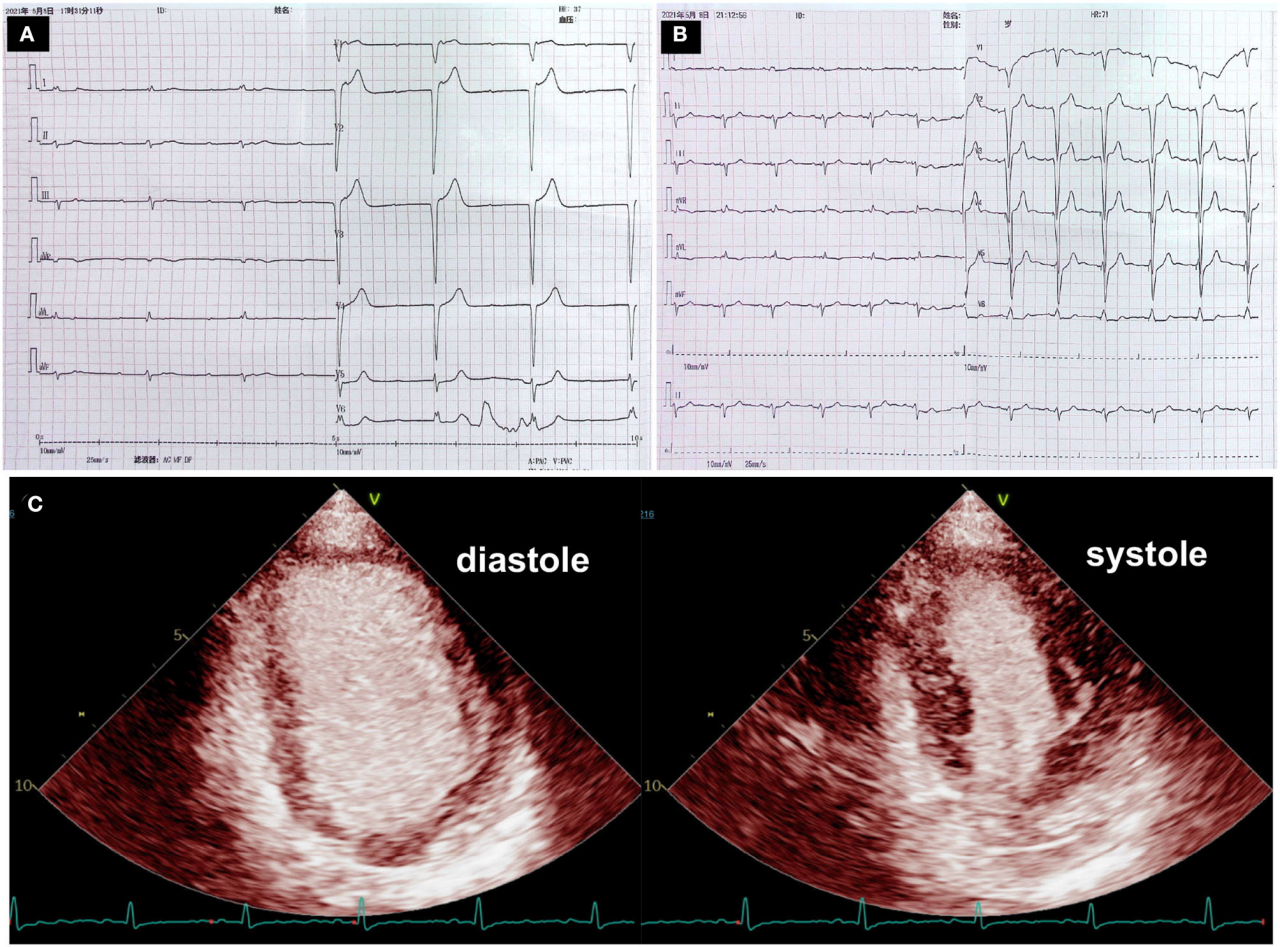
Electrocardiogram and myocardial contrast echocardiography. **(A)** Electrocardiogram at the onset showed complete atrioventricular block with a ventricular escape at a rate of 37 beats per minute. **(B)** Echocardiogram after pacemaker implantation indicated a ventricular pacing rhythm with a wide QRS complex. **(C)** Myocardial contrast echocardiography examinations showed homogenous and equal enhancement intensity in the basal, middle and apical segments.

At 10 h after the second administration of pembrolizumab plus nimotuzumab in the stomatology department, the patient complained of palpitation, faintness, and general fatigue, with no typical symptoms of anginal pectoris. She denied recent prodromal infection. Except for pembrolizumab and nimotuzumab, she did not receive any other medicine. The electrocardiogram (ECG) showed CAVB with a ventricular escape rate at 37 bpm (Figure 1A), which was normal prior to therapy with combined pembrolizumab. The high-sensitivity troponin I (hsTnI) assay showed a moderate elevation of 440 pg/mL (normal <10.4 pg/mL). Serum electrolytes were within normal range, which did not support electrolyte disturbance-induced CAVB.

The patient was then urgently transferred to the cardiology department. Vital signs upon admission indicated heart rate of 37 bpm, blood pressure of 128/54 mmHg, respiratory rate of 18 rpm, temperature of 36°C, and oxygen saturation of 98% in room air. The physical examination revealed a vegetable mass in the right mandibular region. There was no rash, cyanosis, edema or bibasilar rales in the lungs. Except for the abnormal heart rate, cardiovascular examination revealed no other abnormality. She was awake and oriented, with no focal neurologic deficit.

Repeat ECG showed CAVB with a ventricular escape rate at 37 bpm. The initial work-up showed elevated levels of hsTnI of 1,026.5 pg/mL (normal <10.4 pg/mL), creatine kinase (CK) of 510 U/L (normal <165 U/L), CK-MB of 39.8 U/L (normal <5 U/L), and brain natriuretic peptide (BNP) of 2,171 pg/mL (normal <125 pg/mL). Moreover, her white blood cell was 11.8 × 10^9^/μL (normal 4 to 10 × 10^9^/μL), while her C-reactive protein level and erythrocyte sedimentation rate were both within normal limits. Urgent transthoracic echocardiography and chest computed tomography showed unremarkable results.

**Figure 2:**
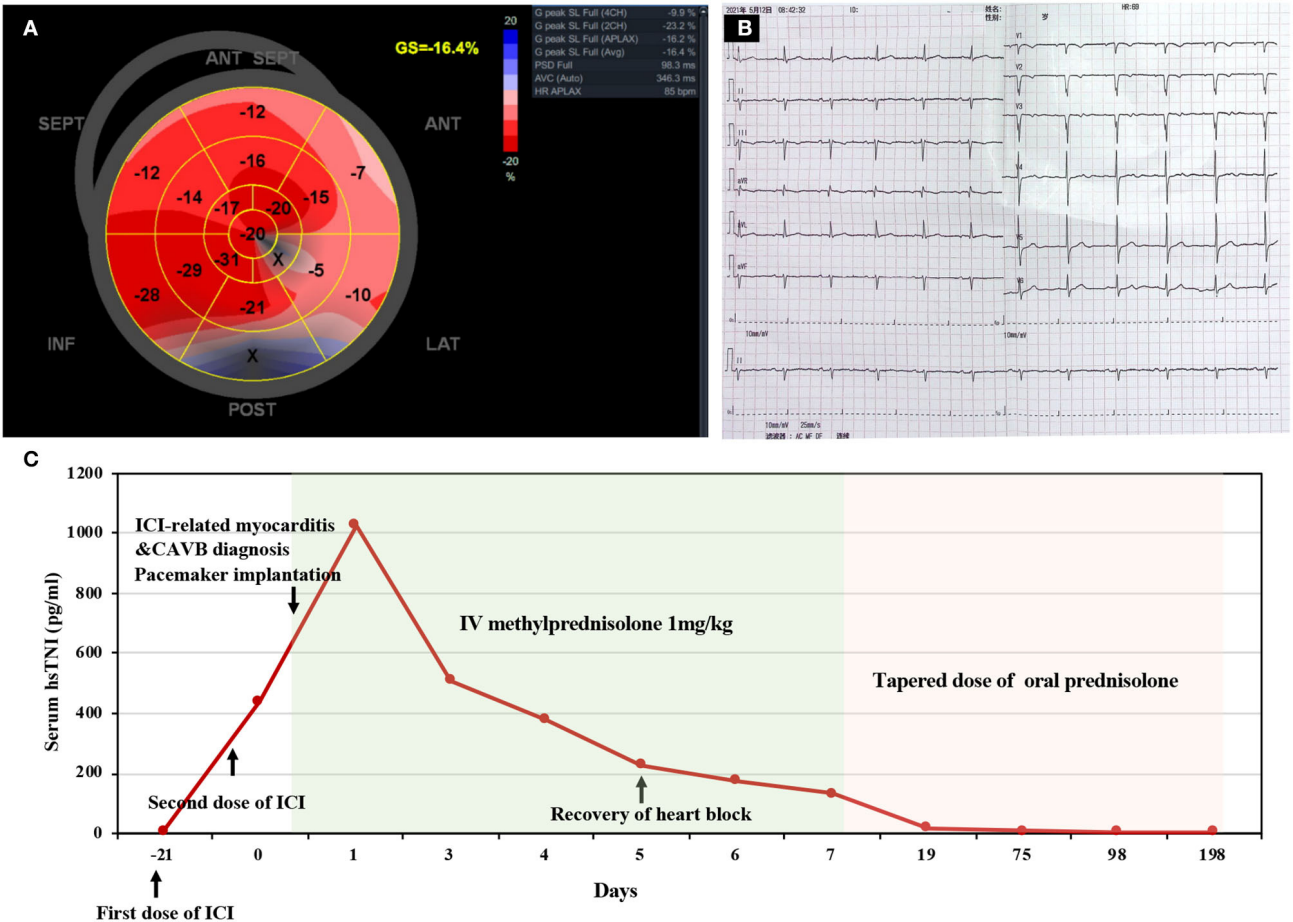
Speckle tracking echocardiography, repeat electrocardiogram, and timeline of disease diagnosis and treatment. **(A)** Global longitudinal strain (GLS) bull's-eye plot showed a slightly decreased GLS of−16.4% and mainly impaired strain in the basal segments of anterior and lateral wall. **(B)** Electrocardiogram on the 5th day of admission showed sinus rhythm with a normal rate of 69 beats per minute. **(C)** Timeline of disease diagnosis and treatment. ICI, immune checkpoint inhibitor; CAVB, complete atrioventricular block; IV, intravenous.

Considering the possibility of myocardial infarction and the potential high risk of cardiac arrest, the patient underwent urgent coronary angiography, which did not reveal any significant stenosis, and a dual-chamber pacemaker was subsequently implanted. The pacemaker was programmed with DDD pacing mode and ECG recorded a ventricular pacing rhythm with a wide QRS complex (Figure 1B). Further diagnostic work-up was performed. Hepatitis B, human immunodeficiency virus (HIV), Epstein–Barr virus, and cytomegalovirus were negative on the basis of serology while thyroid hormone was unremarkable. Therefore, neither viral myocarditis nor thyroid disorder could be considered as the cause of myocarditis. Myocardial contrast echocardiography showed that the perfusion of myocardium was normal (Figure 1C), with a normal left ventricular ejection fraction (LVEF) of 65%. However, speckle tracking echocardiography revealed a slightly decreased left ventricular global longitudinal strain (GLS) of−16.4%. Longitudinal strain was mainly impaired in the basal segments of the anterior and lateral walls (Figure 2A).

Nimotuzumab is marketed and has been administered to over 38,000 patients with limited adverse events (7). Until now, no reports have been published yet regarding myocarditis caused by nimotuzumab. Thus, the strong relationship between the onset time and pembrolizumab therapy suggested that pembrolizumab-induced myocarditis was the most likely diagnosis. In the light of hemodynamic stability as well as advanced age and vulnerability, high-dose glucocorticoids were initiated (1 mg/kg/day intravenous methylprednisolone) without pulse steroids within 24 h after onset. Excitingly, the patient's symptoms rapidly resolved, and repeat ECG revealed the recovery of atrioventricular conduction (Figure 2B) on the 5th day of admission. The treatment response to steroids further supported the diagnosis of pembrolizumab induced myocarditis. The patient was then discharged on the 7th day of admission, and the dosage of glucocorticoids was gradually tapered during the regular outpatient follow-up. The levels of hsTnI, BNP, and CK were gradually reduced to normal limits within 3 weeks after onset. On the 6th month of follow-up, the patient had a normal active life, and the mandibular mass showed no marked enlargement after discontinuation of pembrolizumab therapy. The overview of the clinical course of this patient is illustrated in Figure 2C.

## Literature Review

The largest retrospective study of the WHO VigiBase demonstrated that cardiac irAEs comprise 2,215 (2.09%) of 106,025 irAEs (8). Among the cardiac irAEs, myocarditis was the most common form accounting for 14.1%, followed by pericardial disease (13.6%), conduction abnormalities (6.87%) and stress cardiomyopathy (0.72%) (8). Case reports of ICI-induced CAVB were often assessed to be secondary to myocarditis involving the conduction system. One multicenter trial, involving 30 cases of ICI-associated myocarditis, showed that atrioventricular conduction disorders were observed in 17% of patients with ICI-induced myocarditis (9). Albeit, the clinical characteristics and prognosis of CAVB secondary to ICI-induced myocarditis are less well defined.

We searched PubMed for articles published from inception to March 31th, 2022 in the English language. Search terms included “immune checkpoint” and “myocarditis”. Case reports on human myocarditis concurrent with CAVB caused by ICIs were selected. Totally, we reported 30 cases of ICI-induced myocarditis concurrent with CAVB, with a fatality rate of 60% (see Table 1 for details). The median age of patients was 67 (interquartile range, 63–74) years old and 63% of the patients were male. The median time from ICI administration to the onset of myocarditis was 21 days (interquartile range, 15–26 days). Among these cases, 63% of the patients occurred after the first ICI infusion and 23% occurred after the second ICI infusion. Besides, Most of the patients (87%) had no previous history of cardiovascular diseases and 30% presented EF-reduced heart failure. Of these patients with improved outcomes other than our case, they all received pulse methylprednisolone in the presence or absence of other immunosuppressive therapy. Additionally, 7 out of 12 (58%) surviving patients recovered from conduction disorders after immunosuppressive treatment.

**Table 1:** Published cases of immune checkpoint inhibitor-induced myocarditis concurrent with complete heart block.

**No**	**Ref**.	**Age**	**Sex**	**CVD**	**Tumor**	**ICIs**	**ICIs cycles/days**	**EF%**	**Pulse GC**	**OIT**	**Pacing**	**Outcome**
1	(10)	63	M	–	Melanoma	IP+NI	1/15	50	+	INFI	TP	Death
2	(10)	65	F	–	Melanoma	IP+NI	1/12	73	–	–	–	Death
3	(11)	68	M	–	Sarcoma	IP+NI	1/14	35	+	MYCO	PP	Improved
4	(12)	63	M	HTN	Melanoma	NI	2/21	N	–	–	TP	Death
5	(13)	66	M	–	CMML	IP	1/7	70	+	–	–	Death
6	(14)	76	F	–	Lung cancer	NI	7/98	15	+	IVIG+PLAS+INFI	CRT-D	Improved
7	(15)	69	F	–	Lung cancer	NI	3/38	N	+	–	TP	Improved^**†**^
8	(16)	79	M	–	Gastric cancer	PE	2/35	N	+	IVIG+PLAS+MTX	TP	Death^**†**^
9	(17)	64	F	–	Glioblastoma	NI	2/22	37	+	INFI+ATG+MMF	TP	Improved^**†**^
10	(18)	73	M	–	Lung cancer	PE	1/16	70	+	–	PP	Improved^**†**^
11	(19)	67	M	–	Melanoma	IP+NI	1/16	20	+	ATG	PP	Death
12	(20)	67	F	–	Myeloma	PE	1/16	30	–	INFI	–	Death
13	(21)	67	M	HTN	Melanoma	IP+NI	1/16	N	+	IVIG	–	Death
										INFI		
14	(22)	33	M	–	Lymphoma	NI	8/150	NM	–	MMF+IVIG	–	Death
15	(23)	70	F	–	Thymoma	PE	1/16	N	+	PLAS	PP	Improved
16	(24)	81	M	–	Renal cancer	IP+NI	1/21	62	+	PLAS	TP	Death
17	(25)	88	M	–	Melanoma	NI	1/22	N	+	INFI+MMF	PP	Death
18	(26)	48	F	–	Thymoma	PE	1/13	45	+	INFI	PP	Death
19	(27)	66	M	–	Gastric cancer	NI	1/24	NM	+	IVIG+PLAS	TP	Death
20	(28)	47	F	–	Thymoma	TO	1/28	NM	+	–	TP	Improved^**†**^
21	(29)	57	M	–	Renal cancer	IP+NI	1/12	50	+	ABAT+MMF	PP	Improved
22	(30)	79	F	–	Melanoma	IP+NI	2/25	NM	NM	–	P	Death
23	(31)	73	F	–	Melanoma	NI	1/18	N	–	–	PP	Death
24	(32)	57	M	–	Lung cancer	IP+NI	2/60	N	+	TCZ	P	Improved
25	(33)	70	M	HTN	Renal cancer	IP+NI	1/14	N	+	MMF+PLAS	TP	Death
26	(34)	66	M	–	Lung cancer	SI	2/25	N	+	IVIG	PP	Improved^**†**^
27	(35)	59	M	–	Renal cancer	IP+NI	1/21	N	+	IVIG+PLAS	–	Improved^†^
28	(36)	78	M	CHD	Lung cancer	IP+NI	1/15	45	–	–	–	Death
29	(37)	74	M	–	Gastric cancer	NI	12/240	10	+	IVIG+PLAS	TP	Death
30	ours	80	F	–	HNSCC	PE	2/21	65	–	–	PP	Improved^**†**^

*CVD, cardiovascular disease; ICIs, immune checkpoint inhibitors; EF, ejection fraction; OIT, other immunosuppressive therapy; GC, glucocorticoids; M, male; F, female; HTN, hypertension; CMML, chronic myelomonocytic leukemia; HNSCC, head and neck squamous cell cancer; CHD, coronary heart disease; IP, ipilimumab; NI, nivolumab; PE, pembrolizumab; SI, sintilimab; TO, toripalimab; INFI, infliximab; MMF, mycophenolate mofetil; PLAS, plasmapheresis; IVIG, intravenous immunoglobulins; MTX, methotrexate; ATG, anti-thymocyte globulin; ABAT, abatacept; TP, temporary pacemaker; PP, permanent pacemaker; P, pacemaker; CRT-D, cardiac resynchronization therapy defibrillator; TCZ, tocilizumab; N, normal; NM, not mention. †Recovery to sinus rhythm from complete heart block*.

## Discussion

Cardiotoxicity caused by ICIs is a rare adverse event, while its high mortality rate makes it worthy of further investigation (4). Patients can present with myocarditis, heart failure, arrhythmia, pericardial involvement, myocardial infarction, Takotsubo cardiomyopathy, vasculitis, etc. (2). Among these cardiac irAEs, myocarditis is the most common form with an incidence of 0.27–1.14% (38). The mechanism of ICI-induced myocarditis is still under research. Histopathological analyses of patients with ICI-induced myocarditis revealed that the infiltration of predominant CD8^+^ T lymphocytes and a few macrophages might be the main cause of ICI-induced myocarditiss (39). This inflammation sometimes involves the cardiac conduction system, leading to conduction disorders (39). Therefore, conduction abnormalities frequently present with myocarditis. Furthermore, compared to the mortality rate (50%) of ICI-induced myocarditis in the existing literature, CAVB appeared a trend of increasing the mortality of ICI-induced myocarditis based on the mortality (60%) we reviewed.

The most widely recommended therapy for ICI-associated myocarditis is discontinuation of ICI therapy and administration of high-dose corticosteroids (1–2 mg/kg/day) as soon as possible, which is mainly initiated with pulse methylprednisolone (500–1,000 mg/day) for 3 days and subsequently gradually tapered and off (38). For cases who are unresponsive to corticoids, escalation to other immunosuppressive therapies should be evaluated, frequently involving plasmapheresis, intravenous immunoglobin, mycophenolate, and infliximab (38). Notably, steroid therapy may be accompanied by several adverse effects, particularly in elderly patients, who are vulnerable to adverse outcomes of steroid therapy, notably opportunistic infections. Thus, it is imperative to balance treatment effects of irAEs and adverse effects according to risk stratification. In most cases with cardiac irAEs, myocarditis concomitant with CAVB was clinically categorized as fulminant myocarditis, and pulse methylprednisolone thereby was initiated early after the onset (Table 1). In this report, the patient received high-dose steroids rather than pulse methylprednisolone therapy while clinical and laboratory results were noticeably improved. Importantly, repeated ECG revealed recovery of conduction disorder on the 5th day after its onset. This successful treatment indicated that CAVB does not always portend worse outcomes and may not need aggressive immunosuppressive therapy in such cases. Additionally, suspicion of ICI-induced myocarditis in our patients was raised early, and intravenous corticoids were quickly administrated after ruling out myocardial infarction and viral myocarditis based on medical history and quick-easy examination. As a consequence, early initiation of steroid treatment also contributed to good clinical outcomes.

Despite the limited available data, numerous risk factors for developing cardiac irAEs have been reported, including combination ICI therapy, underlying cardiovascular disease, previous cancer therapy-induced cardiac dysfunction, and underlying autoimmune diseases (38). Additionally, it should be noted that thymic epithelial tumors are associated with a higher incidence of irAEs than other types of cancers (23). Among these patients, cardiac biomarkers and electrocardiograms should warrant intensive monitoring. To our knowledge, we report the first case of CAVB secondary to ICI-induced myocarditis complicated with recurrent HNSCC, and the above-mentioned risk factors were not found in this patient.

Among patients with ICI-induced myocarditis, more than half of patients had a normal LVEF, while they could still develop severe cardiac events (40). GLS has been found as a sensitive marker of cardiac injury with traditional cytotoxic therapy. A recent study on patients with cardiac irAEs showed that each percent reduction in GLS was correlated with a 1.5-fold increase in major adverse cardiovascular events (MACEs) in patients with a reduced EF and a 4.4-fold increase with a preserved EF (41). Importantly, GLS lower than 16% was significantly associated with a higher incidence of MACEs in patients with the preserved LVEF (41). In our case, the patient underwent myocardial contrast (MCE) echocardiography and speckle-tracking echocardiography. Although the slightly decreased GLS with normal MCE demonstrated that myocardial injury was irrelevant to myocardial ischemia, a GLS higher than 16% with a preserved LVEF highly indicated that the patient had a lower risk of subsequent cardiac events based on the existing literature. In support of our speculation, the patient showed an exceedingly good outcome after steroid therapy.

Another interesting finding was that the patient's tumor remained stable during the half-year follow-up after termination of immunotherapy. Of note, current literature has shown that patients with recurrent or metastatic HNSCC who are not amenable to curative therapies have poor survival (6). Despite receipt of aggressive chemotherapy or anti-EGFR monotherapy, the median progression-free survival only ranges from 3 to 4 months in these patients (6). Conceivably, the patient may obtain prolonged disease stabilization from pembrolizumab. However, in the setting of cardiac irAEs, ICIs are frequently recommended for discontinuation.

There are several limitations in the present case report. First, endomyocardial biopsy was not performed due to the patient's refusal given procedural risks. Second, cardiac magnetic resonance (CMR) has been regarded as an gold-standard noninvasive imaging to confirm myocarditis. But our patient refused to further perform CMR due to the improved clinical course. Third, the patient underwent permanent pacing early, but conduction disorder disappeared rapidly after steroid therapy. Although permanent pacing was performed in the majority of the aforementioned cases with concurrence of myocarditis and CAVB (Table 1), we here firstly reviewed that more than half of surviving patients with conduction disorders secondary to ICIs might recover to normal sinus rhythm under immunosuppressive therapy. Consequently, temporary pacemakers should be recommended first. Nevertheless, given the paucity of data on the reversibility of heart block related to ICIs, the indication for and timing of permanent pacemaker placement remains elusive.

In general, ICI-induced myocarditis can be life-threatening and it therefore warrants efforts to increase awareness, facilitate early detection, and initiate prompt intervention. Importantly, CAVB may not always present with fulminant myocarditis and more than 50% of these surviving patients could recover to normal sinus rhythm. When CMR is contraindicated in patients with ICI-related myocarditis, speckle-tracking echocardiography is a reliable and sensitive alternative to CMR for detecting myocardial injury, and GLS may be an important prognostic indicator.

## Data Availability Statement

The original contributions presented in the study are included in the article/supplementary material, further inquiries can be directed to the corresponding author/s.

## Ethics Statement

Written information consent was obtained from the individual(s) for the publication of any potentially identifiable images or data included in this article.

## Author Contributions

LS and CL contributed to data analysis, article drafting, and figure editing. WW provided the history of this patient's tumor and revised the related sections in the manuscript. YC searched the literature. MW collected clinical records. HC revised the manuscript. All authors contributed to the article and approved the submitted version.

## Funding

This study was financially supported by the National Natural Science Foundation of China (No. 81970301).

## Conflict of Interest

The authors declare that the research was conducted in the absence of any commercial or financial relationships that could be construed as a potential conflict of interest.

## Publisher's Note

All claims expressed in this article are solely those of the authors and do not necessarily represent those of their affiliated organizations, or those of the publisher, the editors and the reviewers. Any product that may be evaluated in this article, or claim that may be made by its manufacturer, is not guaranteed or endorsed by the publisher.

## References

[1] Neilan TG Rothenberg ML Amiri-Kordestani L Sullivan RJ Steingart RM Gregory W et al. Myocarditis associated with immune checkpoint inhibitors: an expert consensus on data gaps and a call to action. Oncologist. (2018) 23:874–8. 10.1634/theoncologist.2018-0157 29802220 PMC6156187

[2] Shalata W Abu-Salman A Steckbeck R Mathew Jacob B Massalha I Yakobson A. Cardiac toxicity associated with immune checkpoint inhibitors: a systematic review. Cancers (Basel). (2021) 13:5218. 10.3390/cancers13205218 34680365 PMC8534225

[3] Machiels JP Rene Leemans C Golusinski W Grau C Licitra L Gregoire V et al. Squamous cell carcinoma of the oral cavity, larynx, oropharynx and hypopharynx: EHNS-ESMO-ESTRO Clinical Practice Guidelines for diagnosis, treatment and follow-up. Ann Oncol. (2020) 31:1462–75. 10.1016/j.annonc.2020.07.011 33239190

[4] Ball S Ghosh RK Wongsaengsak S Bandyopadhyay D Ghosh GC Aronow WS et al. Cardiovascular toxicities of immune checkpoint inhibitors: JACC review topic of the week. J Am Coll Cardiol. (2019) 74:1714–27. 10.1016/j.jacc.2019.07.079 31558256

[5] Burtness B Harrington KJ Greil R Soulieres D Tahara M de Castro G Jr. Pembrolizumab alone or with chemotherapy versus cetuximab with chemotherapy for recurrent or metastatic squamous cell carcinoma of the head and neck (KEYNOTE-048): a randomised, open-label, phase 3 study. Lancet. (2019) 394:1915–28. 10.1016/S0140-6736(19)32591-7 31679945

[6] Sacco AG Chen R Worden FP Wong DJL Adkins D Swiecicki P et al. Pembrolizumab plus cetuximab in patients with recurrent or metastatic head and neck squamous cell carcinoma: an open-label, multi-arm, non-randomised, multicentre, phase 2 trial. Lancet Oncol. (2021) 22:883–92. 10.1016/S1470-2045(21)00136-4 33989559 PMC12140401

[7] Bernhard W Barreto K El-Sayed A Gonzalez C Viswas RS Toledo D et al. Pre-clinical study of IRDye800CW-nimotuzumab formulation, stability, pharmacokinetics, and safety. BMC Cancer. (2021) 21:270. 10.1186/s12885-021-08003-3 33711962 PMC7953729

[8] Upadhrasta S Elias H Patel K Zheng L. Managing cardiotoxicity associated with immune checkpoint inhibitors. Chronic Dis Transl Med. (2019) 5:6–14. 10.1016/j.cdtm.2019.02.004 30993259 PMC6450824

[9] Escudier M Cautela J Malissen N Ancedy Y Orabona M Pinto J et al. Clinical features, management, and outcomes of immune checkpoint inhibitor-related cardiotoxicity. Circulation. (2017) 136:2085–7. 10.1161/CIRCULATIONAHA.117.030571 29158217

[10] Johnson DB Balko JM Compton ML Chalkias S Gorham J Xu Y et al. Fulminant myocarditis with combination immune checkpoint blockade. N Engl J Med. (2016) 375:1749–55. 10.1056/NEJMoa1609214 27806233 PMC5247797

[11] Reddy N Moudgil R Lopez-Mattei JC Karimzad K Mouhayar EN Somaiah N et al. Progressive and reversible conduction disease with checkpoint inhibitors. Can J Cardiol. (2017) 33:1335 e13–5. 10.1016/j.cjca.2017.05.026 28822650

[12] Behling J Kaes J Munzel T Grabbe S Loquai C. New-onset third-degree atrioventricular block because of autoimmune-induced myositis under treatment with anti-programmed cell death-1 (nivolumab) for metastatic melanoma. Melanoma Res. (2017) 27:155–8. 10.1097/CMR.0000000000000314 27977496

[13] Berg DD Vaduganathan M Nohria A Davids MS Alyea EP Torre M et al. Immune-related fulminant myocarditis in a patient receiving ipilimumab therapy for relapsed chronic myelomonocytic leukaemia. Eur J Heart Fail. (2017) 19:682–5. 10.1002/ejhf.806 28485549

[14] Frigeri M Meyer P Banfi C Giraud R Hachulla AL Spoerl D et al. Immune checkpoint inhibitor-associated myocarditis: a new challenge for cardiologists. Can J Cardiol. (2018) 34:92 e1–3. 10.1016/j.cjca.2017.09.025 29275889

[15] Fukasawa Y Sasaki K Natsume M Nakashima M Ota S Watanabe K et al. Nivolumab-Induced Myocarditis Concomitant with Myasthenia Gravis. Case Rep Oncol. (2017) 10:809–12. 10.1159/000479958 29070994 PMC5649238

[16] Nasr F El Rassy E Maalouf G Azar C Haddad F Helou J et al. Severe ophthalmoplegia and myocarditis following the administration of pembrolizumab. Eur J Cancer. (2018) 91:171–3. 10.1016/j.ejca.2017.11.026 29287903

[17] Tay RY Blackley E McLean C Moore M Bergin P Gill S et al. Successful use of equine anti-thymocyte globulin (ATGAM) for fulminant myocarditis secondary to nivolumab therapy. Br J Cancer. (2017) 117:921–4. 10.1038/bjc.2017.253 28797029 PMC5625667

[18] Katsume Y Isawa T Toi Y Fukuda R Kondo Y Sugawara S et al. Complete atrioventricular block associated with pembrolizumab-induced acute myocarditis: the need for close cardiac monitoring. Intern Med. (2018) 57:3157–62. 10.2169/internalmedicine.0255-17 29877257 PMC6262691

[19] Jain V Mohebtash M Rodrigo ME Ruiz G Atkins MB Barac A. Autoimmune myocarditis caused by immune checkpoint inhibitors treated with antithymocyte globulin. J Immunother. (2018) 41:332–5. 10.1097/CJI.0000000000000239 29965858

[20] Martinez-Calle N Rodriguez-Otero P Villar S Mejias L Melero I Prosper F et al. Anti-PD1 associated fulminant myocarditis after a single pembrolizumab dose: the role of occult pre-existing autoimmunity. Haematologica. (2018) 103:e318–21. 10.3324/haematol.2017.185777 29650641 PMC6029537

[21] Saibil SD Bonilla L Majeed H Sotov V Hogg D Chappell MA et al. Fatal myocarditis and rhabdomyositis in a patient with stage IV melanoma treated with combined ipilimumab and nivolumab. Curr Oncol. (2019) 26:e418–21. 10.3747/co.26.4381 31285688 PMC6588051

[22] Charles J Giovannini D Terzi N Schwebel C Sturm N Masson D et al. Multi-organ failure induced by Nivolumab in the context of allo-stem cell transplantation. Exp Hematol Oncol. (2019) 8:8. 10.1186/s40164-019-0132-2 30963019 PMC6437980

[23] Szuchan C Elson L Alley E Leung K Camargo AL Elimimian E et al. Checkpoint inhibitor-induced myocarditis and myasthenia gravis in a recurrent/metastatic thymic carcinoma patient: a case report. Eur Heart J Case Rep. (2020) 4:1–8. 10.1093/ehjcr/ytaa051 32617460 PMC7319805

[24] Hardy T Yin M Chavez JA Ivanov I Chen W Nadasdy T et al. Acute fatal myocarditis after a single dose of anti-PD-1 immunotherapy, autopsy findings: a case report. Cardiovasc Pathol. (2020) 46:107202. 10.1016/j.carpath.2020.107202 32062109

[25] Giancaterino S Abushamat F Duran J Lupercio F DeMaria A Hsu JC. Complete heart block and subsequent sudden cardiac death from immune checkpoint inhibitor-associated myocarditis. HeartRhythm Case Rep. (2020) 6:761–4. 10.1016/j.hrcr.2020.07.015 33101950 PMC7573344

[26] Portoles Hernandez A Blanco Clemente M Escribano Garcia D Velasco Calvo R Nunez Garcia B Oteo Dominguez JF et al. Checkpoint inhibitor-induced fulminant myocarditis, complete atrioventricular block and myasthenia gravis-a case report. Cardiovasc Diagn Ther. (2021) 11:1013–9. 10.21037/cdt-21-147 34527524 PMC8410500

[27] Komatsu M Hirai M Kobayashi K Hashidate H Fukumoto J Sato A et al. A rare case of nivolumab-related myasthenia gravis and myocarditis in a patient with metastatic gastric cancer. BMC Gastroenterol. (2021) 21:333. 10.1186/s12876-021-01904-4 34445963 PMC8393464

[28] Luo YB Tang W Zeng Q Duan W Li S Yang X et al. Case Report: The neuromusclar triad of immune checkpoint inhibitors: a case report of myositis, myocarditis, and myasthenia gravis overlap following toripalimab treatment. Front Cardiovasc Med. (2021) 8:714460. 10.3389/fcvm.2021.714460 34485412 PMC8415306

[29] Jespersen MS Fano S Stenor C Moller AK. A case report of immune checkpoint inhibitor-related steroid-refractory myocarditis and myasthenia gravis-like myositis treated with abatacept and mycophenolate mofetil. Eur Heart J Case Rep. (2021) 5:ytab342. 10.1093/ehjcr/ytab342 34870082 PMC8637790

[30] Barham W Guo R Park SS Herrmann J Dong H Yan Y. Case report: simultaneous hyperprogression and fulminant myocarditis in a patient with advanced melanoma following treatment with immune checkpoint inhibitor therapy. Front Immunol. (2020) 11:561083. 10.3389/fimmu.2020.561083 33603731 PMC7884751

[31] Wang F Sun X Qin S Hua H Liu X Yang L et al. A retrospective study of immune checkpoint inhibitor-associated myocarditis in a single center in China. Chin Clin Oncol. (2020) 9:16. 10.21037/cco.2020.03.08 32279526

[32] Doms J Prior JO Peters S Obeid M. Tocilizumab for refractory severe immune checkpoint inhibitor-associated myocarditis. Ann Oncol. (2020) 31:1273–5. 10.1016/j.annonc.2020.05.005 32425357 PMC7229714

[33] Gonzalez-Ferrero T Vargas-Osorio K Gonzalez-Juanatey JR. Fulminant myocarditis with myositis after treatment with immune checkpoint inhibitors. Med Clin (Barc). (2022) 158:140–1. 10.1016/j.medcli.2021.04.014 34294442

[34] Xing Q Zhang ZW Lin QH Shen LH Wang PM Zhang S et al. Myositis-myasthenia gravis overlap syndrome complicated with myasthenia crisis and myocarditis associated with anti-programmed cell death-1 (sintilimab) therapy for lung adenocarcinoma. Ann Transl Med. (2020) 8:250. 10.21037/atm.2020.01.79 32309397 PMC7154453

[35] Yanase T Moritoki Y Kondo H Ueyama D Akita H Yasui T. Myocarditis and myasthenia gravis by combined nivolumab and ipilimumab immunotherapy for renal cell carcinoma: a case report of successful management. Urol Case Rep. (2021) 34:101508. 10.1016/j.eucr.2020.101508 33318935 PMC7726655

[36] Vartanov A Kalotra A Varughese J Gautam S Kandel S Hosmer W. Immunotherapy-associated complete heart block in a patient with NSCLC: a case report and literature review. Respir Med Case Rep. (2021) 33:101390. 10.1016/j.rmcr.2021.101390 33786301 PMC7994778

[37] Naganuma K Horita Y Matsuo K Miyama Y Mihara Y Yasuda M et al. An autopsy case of late-onset fulminant myocarditis induced by nivolumab in gastric cancer. Intern Med. (2022). 10.2169/internalmedicine.9161-21 PMC9593165 35249925

[38] Hu JR Florido R Lipson EJ Naidoo J Ardehali R Tocchetti CG et al. Cardiovascular toxicities associated with immune checkpoint inhibitors. Cardiovasc Res. (2019) 115:854–68. 10.1093/cvr/cvz026 30715219 PMC6452314

[39] Tajiri K Ieda M. Cardiac complications in immune checkpoint inhibition therapy. Front Cardiovasc Med. (2019) 6:3. 10.3389/fcvm.2019.00003 30729114 PMC6351438

[40] Mahmood SS Fradley MG Cohen JV Nohria A Reynolds KL Heinzerling LM et al. Myocarditis in patients treated with immune checkpoint inhibitors. J Am Coll Cardiol. (2018) 71:1755–64. 10.1016/j.jacc.2018.02.037 29567210 PMC6196725

[41] Awadalla M Mahmood SS Groarke JD Hassan MZO Nohria A Rokicki A et al. Global longitudinal strain and cardiac events in patients with immune checkpoint inhibitor-related myocarditis. J Am Coll Cardiol. (2020) 75:467–78. 10.1016/j.jacc.2019.11.049 32029128 PMC7067226

